# Editorial: Recent advances in liquid biopsy in colorectal cancer

**DOI:** 10.3389/fonc.2022.1099526

**Published:** 2022-12-07

**Authors:** Claudia Cardone, Alfonso De Stefano, Antonio Avallone

**Affiliations:** Experimental Clinical Abdominal Oncology Unit, Istituto Nazionale Tumori-IRCCS-Fondazione G. Pascale, Naples, Italy

**Keywords:** colorectal cancer, liquid biopsy, ctDNA, biomarkers, RAS

Liquid biopsy is a test performed on a blood sample to detect circulating cancer cells (CTCs) or DNA fragments originating from tumor cells (ctDNA). The National Cancer Institute’s Dictionary of Cancer Terms provides this definition without accounting for the wide variety of tumor cell products that may act as tumor biomarkers. In addition, aside from blood, other body fluids can also serve as potential liquid biopsy sources. Indeed, liquid biopsy represents an area of active research. In the last decades, the development of advanced technologies for detecting blood-based, tumor-specific biomarkers has improved tumor molecular characterization, treatment decisions and follow-up strategies.

Our Research Topic explored *“Recent Advances in Liquid Biopsy in Colorectal Cancer”*. Specifically, the issue includes seven multidisciplinary manuscripts focusing on multiple topics related to the use and the implementation of liquid biopsy: 1) Detection of minimal residual disease (MRD) 2) Monitoring of tumor response and assessment of mechanisms of resistance during anti-epidermal growth factor receptorederr (EGFR) treatment 3) Exploring potential novel biomarkers 4) Current use in clinical practice (**Figure 1**).

**Figure 1:**
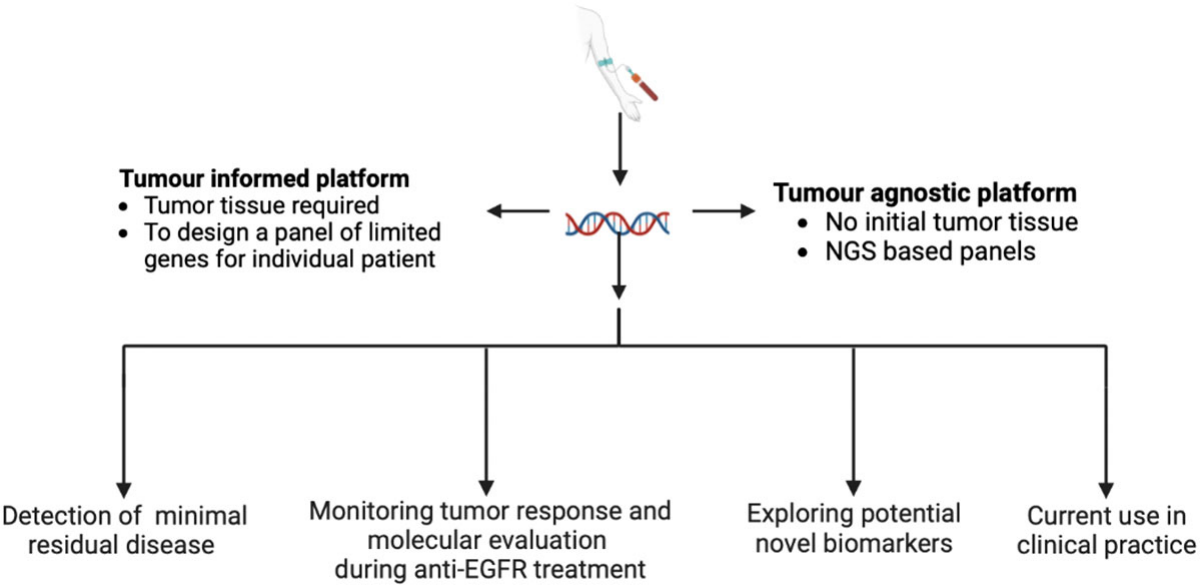
Implementation of liquid biopsy in the management of colorectal cancer.

Multiple studies have shown that ctDNA levels in the early stages can predict MRD in several malignancies, including CRC. In this issue, two studies confirmed the potential use of ctDNA in early CRC and locally advanced rectal cancer. Grancher et al. retrospectively evaluated the impact of ctDNA status after curative surgery in patients with stage II CRC enrolled in the PROGIGE 13 trial with available paired tumour and blood samples. Postoperative ctDNA was detected in 9.0% of the samples and was associated with a higher rate of tumor recurrence and with worse overall survival.

Response after neoadjuvant chemoradiation was monitored with longitudinal liquid biopsies in patients with locally advanced rectal cancer (LARC) in the study presented by Roesel et al. ctDNA assessment by next-generation sequencing (NGS) technology identified patients with LARC who obtainined poor pathological response after neoadjuvant chemoradiation (Tumor Regression Grade 4). Of note, ctDNA levels did not predict complete pathological response (Tumor Regression Grade 1), failing to identify patients who could undergo a non-operative management (NOM).

Liquid biopsy is also helpful to select patients with RAS and BRAF wild-type mCRC, candidates to a rechallenge treatment with anti-EGFR agents. Mechanisms of acquired resistance, often consisting of mutations in the downstream pathways, such as the mitogen-activated protein kinase (MAPK) signalling, can be easily detected by liquid biopsy techniques.

Chennamadhavuni and Kasi presented data from a small series of patients with RAS and BRAF wild-type mCRC, who received prior anti-EGFR therapy and were assessed with ctDNA-based testing. The NGS analysis reported *KRAS, NRAS, EGFR* extracellular domain, and *BRAF* mutations as mechanisms of acquired resistance to anti-EGFR treatment. Longitudinal ctDNA study revealed that variant allele fraction (VAF) percentage varied and decreased over time in relation to the timing of prior EGFR exposure and to the suspension of non-EGFR-based therapy, namely as EGFR holiday period.

In addition, liquid biopsy represents a potential biomarker for prognostic evaluation and for dynamic monitoring of response to anti-EGFR treatment. Yang et al. longitudinally analyzed plasma samples, collected at baseline and at specific time-points, from a cohort of patients with mCRC treated with anti-EGFR-based therapies. The authors observed that a decrease in ctDNA levels predicted both clinical and radiological response assessed by carbohydrate antigen 19-9, carcinoembryonic antigen (CEA) and by computed tomography.

Liquid biopsy offers a manageable method of detecting novel biomarkers. Currently, blood-based sampling is the preferred liquid biopsy technique, representing a standardized and minimally invasive procedure. In the study of Hu et al., serological antigen identification by cDNA expression cloning (SEREX), identified BRCA1-Associated ATM Activator 1 (BRAT1) and WD Repeat Domain 1-antibody (WDR1-Ab) as potential biomarkers of atherosclerosis. Interestingly, BRAT1 was found upregulated in GI cancers, including CRC, suggesting a biological association between the two diseases.

Nevertheless, other body fluids, such as urine, saliva, cerebrospinal fluid, pleural fluid, and peritoneal fluid, may contain tumor biomarkers detectable through a liquid biopsy. Accordingly, Pu et al. supported the clinical significance of ctDNA detection in ascites in patients with peritoneal carcinomatosis. In addition, ctDNA levels were associated with tumor burden and clinical outcome in patients with peritoneal carcinomatosis undergoing hyperthermic intraperitoneal chemotherapy (HIPEC).

Ultimately, liquid biopsy has emerged as a valuable tool to optimize treatment strategies and implement the molecular characterization of patients with CRC. However, despite the potential advantages, it is not yet routinely adopted in clinical practice.

A retrospective study conducted by Fischer et al. examined ctDNA testing in two German comprehensive cancer centers for patients with CRC. Discordance between tissue and liquid analyses was observed in almost 30% of the evaluated samples. In particular, some patients with baseline RAS mutations showed RAS wild type status in subsequent liquid biopsies, possibly defining a distinct molecular entity called NEORAS wild type. According to the authors, despite its potential benefits, liquid biopsy is not routinely used in everyday clinical practice. This is mostly due to method diversity, lack of standardization, and reimbursement restrictions.

For all these reasons, the development of well-designed prospective clinical trials is crucial to incorporate liquid biopsy into clinical guidelines for the treatment of patients with CRC and to implement its use in clinical practice.

## Author contributions

CC drafted the manuscript. AS and AA critically reviewed the manuscript and approved it for publication.

